# Formulation Optimization and Performance Prediction of Red Mud Particle Adsorbents Based on Neural Networks

**DOI:** 10.3390/molecules29050970

**Published:** 2024-02-22

**Authors:** Longjiang Li, Yalan Wang, Wenyuan Wang

**Affiliations:** 1 Mining College, Guizhou University, Guiyang 550025, China; 15809275281@163.com (Y.W.); 18744774725@163.com (W.W.); 2 National & Local Joint Laboratory of Engineering for Effective Utilization of Regional Mineral Resources from Karst Areas, Guiyang 550025, China; 3 Guizhou Key Laboratory of Comprehensive Utilization of Non-Metallic Mineral Resources, Guiyang 550025, China

**Keywords:** back-propagation neural networks, red mud, optimization formulations, wastewater treatment

## Abstract

Red mud (RM), a bauxite residue, contains hazardous radioactive wastes and alkaline material and poses severe surface water and groundwater contamination risks, necessitating recycling. Pretreated RM can be used to make adsorbents for water treatment. However, its performance is affected by many factors, resulting in a nonlinear correlation and coupling relationship. This study aimed to identify the best formula for an RM adsorbent using a mathematical model that examines the relationship between 11 formulation types (e.g., pore-assisting agent, component modifier, and external binder) and 9 properties (e.g., specific surface area, wetting angle, and Zeta potential). This model was built using a back-propagation neural network (BP) based on single-factor experimental data and orthogonal experimental data. The model trained and predicted the established network structure to obtain the optimal adsorbent formula. The RM particle adsorbents had a pH of 10.16, specific surface area (BET) of 48.92 m^2^·g^−1^, pore volume of 2.10 cm^3^·g^−1^, compressive strength (ST) of 1.12 KPa, and 24 h immersion pulverization rate (*η_m_*) of 3.72%. In the removal of total phosphorus in flotation tailings backwater, it exhibited a good adsorption capacity (Q) and total phosphorous removal rate (*η*) of 48.63 mg·g^−1^ and 95.13%, respectively.

## 1. Introduction

Red mud (RM) is a bauxite residue produced in the alumina industry. On average, 1–1.5 tons of RM are produced for every ton of alumina produced [1,2]. The abandoned RM not only occupies a large area of land but also contains thorium, potassium, and other radioactive elements [3,4], which are hazardous solid wastes. In addition, the RM is an alkaline material and the alkali dissolves in the rainwater and contaminates the surface water and groundwater, causing severe environmental pollution [5]. Therefore, multichannel and massive recycling of RM resources is necessary. RM has a small particle diameter and a pore frame structure. It has a substantially larger pore ratio than typical soil with a large specific surface area. Hematite, goethite, and other minerals can also be found in the RM [6,7,8]. Pretreated RM can adsorb radioactive substances such as Co^2+^ and Sr^2+^ [9], heavy metal ions such as Cu^2+^ and Pb^2+^ [10], non-metallic hazardous compounds such as PO_4_^3−^ and As^3+^, and some organic contaminants [11,12]. It can also be used for wastewater decolorization and clarity [13,14] and for calcination in the range of 500 °C–800 °C to produce macroporous iron and carbon-combined calcined and carbon-calcined red mud. The testing results show that RM calcined with iron and carbon additives achieved the highest U adsorption capacity (59.45 mg·g^−1^) under proper calcination temperature (i.e., 600 °C), pH of 2.5, and optimum reaction conditions. Lyu, F, et al. [15] used a hydrothermal approach to modify RM by adsorbing Pb(II) ions in aqueous solutions using colloidal silica and sodium hydroxide under mild conditions. According to the experimental results, the saturated adsorption capacity of the modified RM for Pb(II) ion is ~564.97 mg·g^−1^, and the Langmuir constant KL is 0.23, which suggests that the adsorption process is favorable.

Using RM as the primary adsorbent, a spherical RM particle adsorbent with a double-layer structure was prepared in this study. The RM particle adsorbents have a high adsorption capacity and strong mechanical properties for phosphorus in phosphate ore flotation wastewater. The experimental results show that the composition of the raw material has a great influence on the adsorption efficiency of the adsorbent. Many factors affect the performance of the adsorbent, since most parameters of the adsorbent change nonlinearly, traditional methods are inadequate to determine the optimal formulation for adsorbents. The current material formulation optimization methods mainly include the orthogonal experimental methods [16] and the response surface methodology (RSM) [17]. For complex systems with multivariable, nonlinear, and highly coupled variables, the traditional data analysis methods cannot satisfy the requirements. In 1986, Romelhart and McClelland proposed the error back-propagation (BP) algorithm. Since the training of the multilayer feedforward network often uses this algorithm, the multilayer feedforward network is often called the BP network. Many scientists [18,19,20] developed removal rate and adsorption capacity models using artificial neural network methods. The results show that these models can predict the removal rate and adsorption capacity of ferricyanide using active RM by changing input variables. Jie [21] used RSM and artificial neural network (ANN) to study the effects of HCl concentration, temperature, and time on the adsorption of phosphorus by activated bauxite; the results show that the prediction accuracy of the artificial neural network is better than that of RSM. Therefore, the BP neural network can be well applied to multifactor formulation optimization, realizing the transformation from a linear model to a nonlinear model, and can predict the experimental results well.

Research showed that many factors affect the adsorption of RM adsorbent, including the preparation methods, raw material formulation, drug system, operating conditions, and adsorption state. By establishing a multi-input and multi-output comprehensive evaluation model based on a BP neural network model, the sensitive factors of the adsorption process are obtained; this allows a comprehensive evaluation of the adsorption performance. With regard to the parameter optimization for adsorption, it is a dynamic process with many influencing parameters, using a neural network to optimize the parameters can greatly reduce the number of tests, modify the test’s precision, and improve the efficiency.

In the adsorption theory of adsorbents, generally good adsorbents, along with the research of adsorption, have to be considered for desorption, regeneration, and utilization [22]. Judging the combined effect of the influencing factors on the RM adsorbent is mainly based on the following: the adsorbent must have a large specific surface area, high adsorption capacity and removal amount, and a certain degree of water resistance strength, be capable of regeneration and reuse, cheap, and not pollute the environment. Therefore, in this paper, fly ash (FA) is used to adjust the adsorption capacity, hydrogen peroxide foaming agent is used to adjust the specific surface area, and surfactant is used to adjust the wettability. In contrast, cement and sodium sulfate are used to enhance the mechanical properties of red mud absorbent, so as to make the pulverization rate of red mud in water lower, thus achieving the purpose of reuse. This paper presents an integrated computational intelligence approach based on a hybrid strategy of neural networks and a multiobjective evolutionary algorithm. The BP neural network was used to establish a multi-input and multi-output neural network model to optimize the formulation. The experimental data were trained to obtain a neural network model that reflected the nonlinear mapping relationship between the parameter vector space and target vector space during parameter optimization. The trained neural network model was embedded into the multiobjective evolutionary algorithm, which is used as the individual fitness evaluation function in the evolutionary process. Hence, the multiobjective evolutionary algorithm could be directly applied in product parameter optimization design.

## 2. Results and Analysis

### 2.1. Network Training and Testing

**Figure 1:**
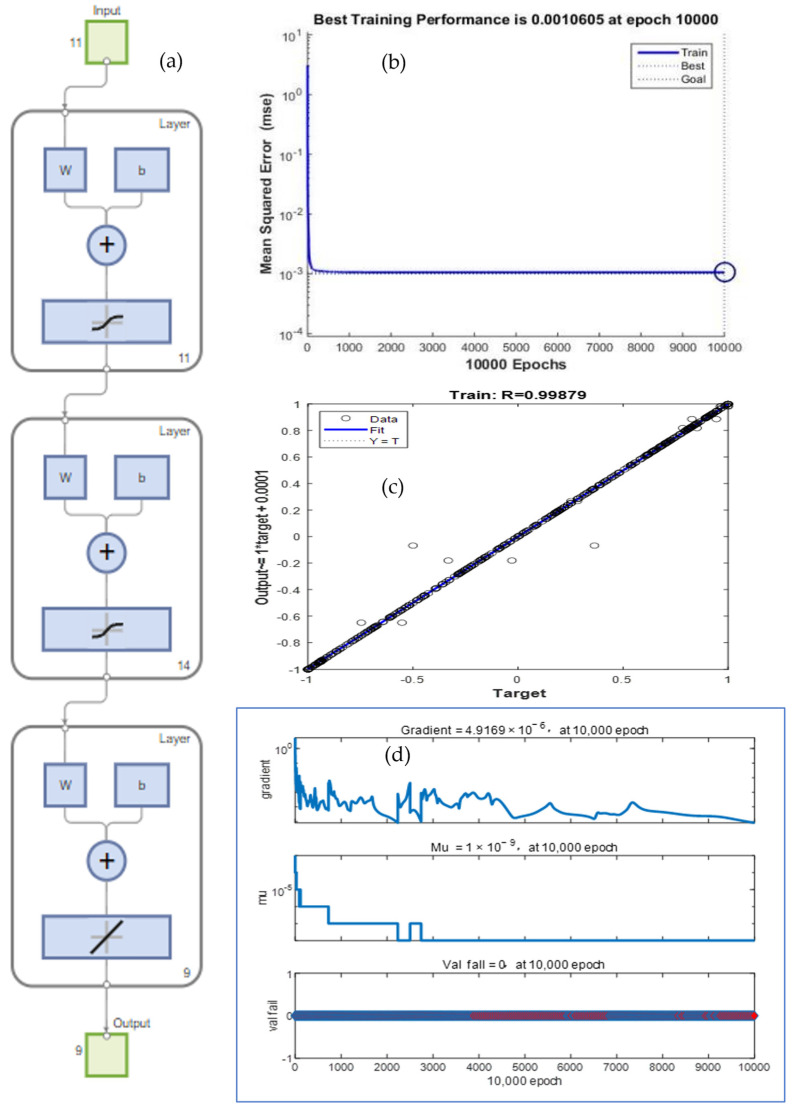
Training process: (**a**) 11×14×9 three-layer BP neural network model; (**b**) epochs–MSE curve; (**c**) target–output curve and (**d**) epochs–gradient, epochs–mu, and epochs–val fail curves.

In order to obtain a highly accurate BP neural network model, we configured the parameters and input training data pairs to train the network; a partial of the training input and output data are given in Table 1 and Table 2. The network training processing is shown in Figure 1, where Figure 1a is an 11×14×9 three-layer BP neural network model, Figure 1b shows the epochs and MSE curve, Figure 1c,d shows the target–output and the epochs–gradient, epochs–mu, and epochs–val fail curves. For a training time of 4 min and 30 s at 10,000 epochs, the network error converged to 0.0010605, and the training correlation coefficient R^2^ = 0.99879, gradient = 4.9169 × 10^−6^, mu = 1 × 10^−9^, val fail = 0, and RMSE = 0.0235, which indicates that the model is not overfitted, the projected accuracy was achieved.

**Figure 2:**
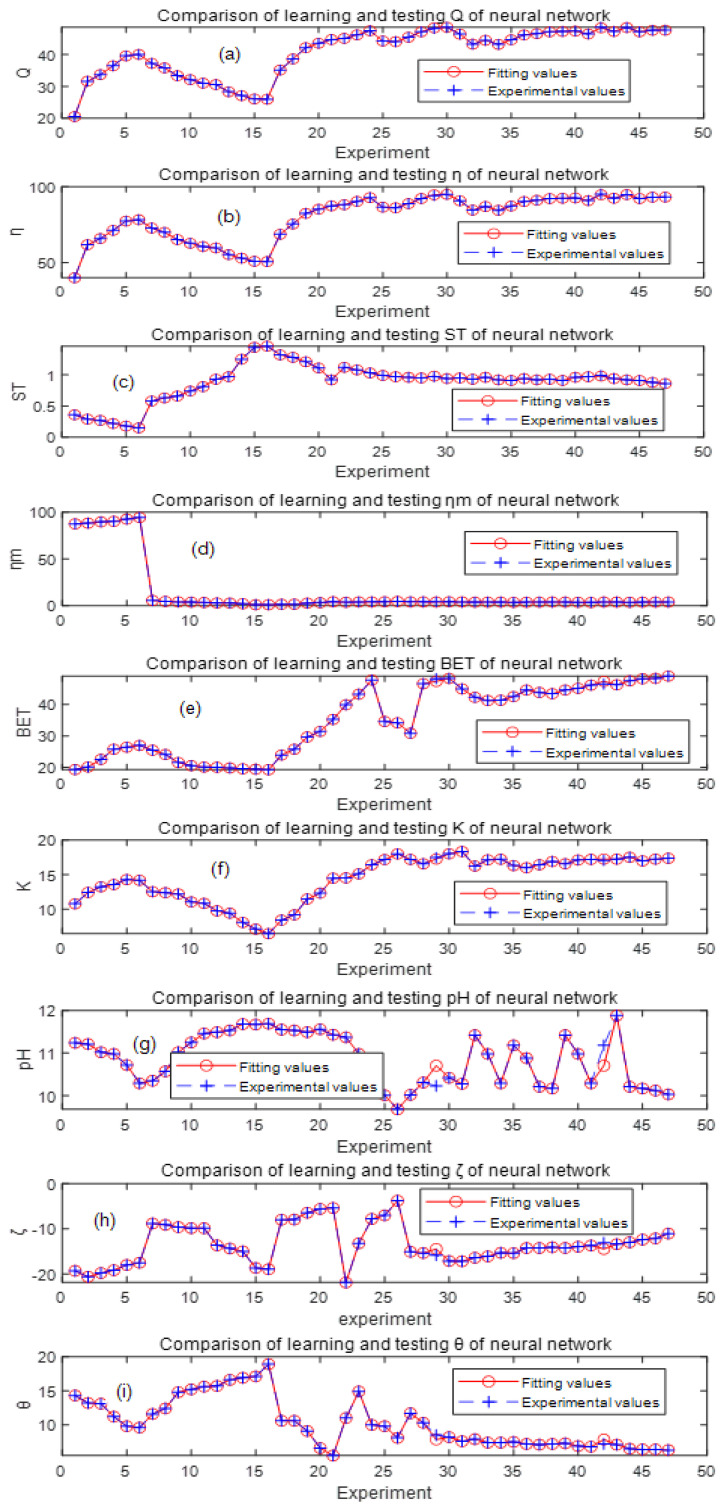
Comparison of experimental and testing values of the output parameters: (**a**) adsorption capacity (Q); (**b**) removal rate (*η*); (**c**) compressive strength (ST); (**d**) immersion pulverization rate ($\eta_{m}$); (**e**) specific surface area (BET); (**f**) water absorption rate (K); (**g**) pH; (**h**) Zeta potential (ζ); and (**i**) wetting angle (θ).

The trained network was tested using testing samples; a partial of the testing input and output data are given in Table 3 and Table 4, and the corresponding experimental values are given in Table 5. By comparing the testing values and the experimental values in Table 4 and Table 5, the MSE = 0.0011, RMSE = 0.0326, and R^2^ = 0.99892. A comparison of testing values and experimental values of the nine output parameters of the network is shown in Figure 2a–i. The RMSE of each output parameter is shown in the last row of Table 4; it can be seen that the fitting value is consistent with the experimental value, the network error is modest, and the convergence is good, so the BP neural network model can be used for parameter optimization and prediction [22].

### 2.2. Performance Analysis of RM Particle Adsorbents

**Figure 3:**
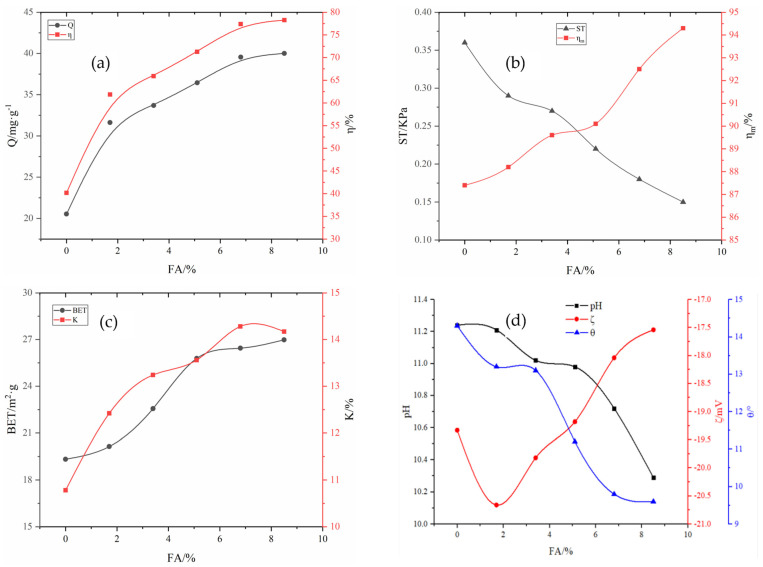
Influence of FA on various adsorbent properties: (**a**) adsorption capacity (Q) and removal rate (*η*); (**b**) compressive strength (ST) and immersion pulverization rate ($\eta_{m}$); (**c**) specific surface area (BET) and water absorption rate (K); (**d**) pH, wetting angle $\left(\theta \right),$ and Zeta potential (ζ).

From the above analysis, the established BP neural network has good accuracy and can be used to calculate and analyze each influencing factor on the performance of red mud adsorbent [22]. According to the method in Section 3.4.4 and the data thresholds in Table 6, we carried out a multipoint calculation and prediction of FA, A2C, HPMC, H_2_O_2_, HCl, and SDBS, respectively. Firstly, we divided the thresholds of each input factor into five equal parts, took five single-factor experimental points, and calculated their adsorption performance parameters, which were expressed as data points. Then, according to the thresholds of the input factors, we took 150 points from the smallest to largest and then calculated its adsorption performance parameters, fitting the synthesis curve. We studied the adsorption properties such as Q, *η*, ST, $\eta_{m}$, BET, K, pH, θ, and ζ to find the optimum dosage of chemicals according to the performance. Subsequently, we prepared the qualified adsorbent of red mud particles according to the optimum dosage of chemical formulation and further verified its performance. For example, when we study the effect of FA on the performance of the adsorbent, we can observe that among the whole formulation, the allowable addition of FA is wt 8.5%, as shown in Table 6. We first set the value of other agents and examine their average points at 1.7%, 3.4%, 5.1%, 6.8%, and 8.5%. We input these values into the trained BP neural network to calculate the performance value of 5 points. Subsequently, we calculate the performance value from 0 to 8.5%, considering 150 points for point-by-point calculation, and then fit the curve, see Figure 3, to find the best dosage point. The latter influencing factors are calculated in the same way, and no more examples are given.

#### 2.2.1. Effect of FA on Adsorbent Properties

Fixing the amount of other agents, according to the threshold interval [0 wt 8.5%] of fly ash in Table 6, from small to large in accordance with a certain step size, we take 150 points into the optimized BP neural network for the calculation and inverse normalization and fit the synthetic curve, as shown in Figure 3. As shown in Figure 3a–d, after adding FA to RM, the removal rate of total phosphorus gradually increased, but FA reduced the adhesion and strength of the adsorbent. When the amount of FA added was 8.5%, the removal rate of total phosphorus reached 78.27%, while the pulverization rate reached 94.30%. As can be seen, the specific surface area and water absorption increased with an increase in FA content. As the FA content increased to 8.5%, the specific surface area increased to 26.98 m^2^·g^−1^, and the water absorption increased to 14.17%. FA is an acidic secondary ash with fine particles and a large specific surface area [23]; the addition of FA results in a smaller wetting angle, enhanced hydrophilicity, and reduced pH value. Furthermore, the Zeta potential decreased first and then increased in the positive direction, which is not conducive to the stability of a solution system but is beneficial to the adsorption of anions [24,25].

#### 2.2.2. Effect of Enhanced and Anti-Chalking Agents on the Adsorbent Properties

**Figure 4:**
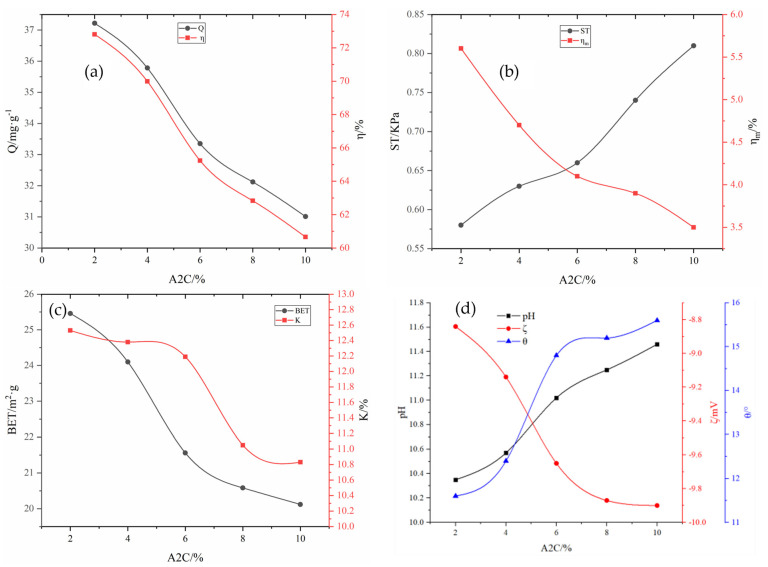
Influence of A2C on various adsorbent properties: (**a**) adsorption capacity (Q) and removal rate (*η*); (**b**) compressive strength (ST) and immersion pulverization rate ($\eta_{m}$); (**c**) specific surface area (BET) and water absorption rate (K); (**d**) pH, wetting angle $\left(\theta \right),$ and Zeta potential (ζ).

Fixing the amount of other agents, according to the threshold interval from wt 2% to wt 8.5% of A2C in Table 6, from small to large in accordance with a certain step size, we take 150 points into the optimized BP neural network for the calculation and inverse normalization and fit the synthetic curves as shown in Figure 4a–d. The enhanced and anti-chalking agents used in this work are mainly the combined reagent A2C/HPMC/Na_2_SiO_4_/KH-602. The influence of A2C on the performance of the adsorbent when the dose of fixed coupling agent KH-602 was 0.1% is shown in Figure 4. When the dose of A2C was increased from 2% to 10%, the compressive strength of the adsorbent increased from 0.58 KPa to 0.81 KPa, and the pulverization rate decreased from 5.60% to 3.50%. Meanwhile, the adsorption capacity of A2C for total phosphorus decreased from 37.22 mg·g^−1^ to 31.01 mg·g^−1^, and the removal rate decreased from 72.81% to 60.66%; with additional A2C, the specific surface area of the adsorbent and water absorption rate decrease. Adding A2C to the adsorbent increases the pH and the wetting angle and weakens the hydrophilicity, while also increasing the Zeta potential in a negative direction and weakening anion adsorption.

**Figure 5:**
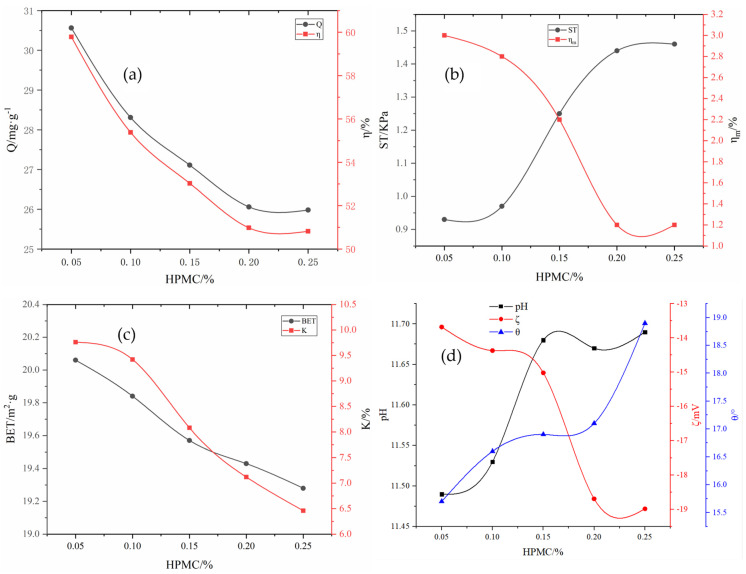
Influence of HPMC on various adsorbent properties: (**a**) adsorption capacity (Q) and removal rate (*η*); (**b**) compressive strength (ST) and immersion pulverization rate ($\eta_{m}$); (**c**) specific surface area (BET) and water absorption rate (K); (**d**) pH, wetting angle $\left(\theta \right),$ and Zeta potential (ζ).

By fixing the additional amount of A2C to 8% and adding 2% Na_2_SiO_4_ and 0.1% KH-602, a certain amount of HPMC was added to further adjust the mechanical properties of the particle adsorbents. The influence of HPMC on the adsorbent properties is shown in Figure 5a–d. Under identical conditions, the addition of HPMC had a great effect on adsorbent performance. With the increase in the amount of HPMC added, the adsorption capacity of the adsorbent for total phosphorus decreased. When the dose of HPMC increased from 0.05% to 0.25%, the adsorption capacity of total phosphorus decreased from 30.56 mg·g^−1^ to 25.98 mg·g^−1^, and the removal rate decreased from 59.78% to 50.82%. But the strength increased from 0.93 kPa to 1.46 kPa, and the pulverization rate of the particles decreased significantly. When the dose of HPMC was 0.25%, the 24 h pulverization rate decreased to 1.2%. The effects of HPMC on the specific surface area and water absorption rate were similar to those of A2C. HPMC has little influence on pH, but it does raise the wetting angle, weakens the hydrophilicity, increases the Zeta potential in a negative direction, and develops the system in a stable direction, which is unfavorable adsorption.

#### 2.2.3. The Effect of Non-Thermal Pore-Forming Agent H_2_O_2_ on Adsorbent Properties

**Figure 6:**
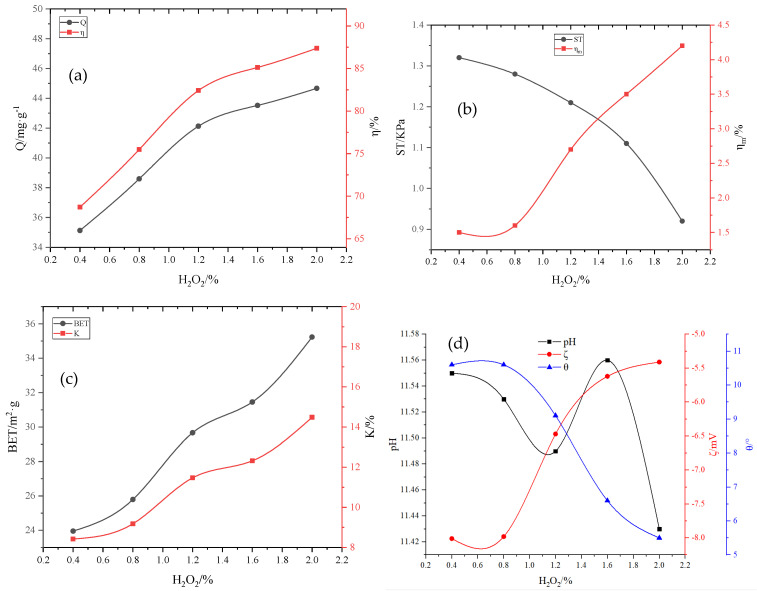
Influence of H_2_O_2_ on various adsorbent properties: (**a**) adsorption capacity (Q) and removal rate (*η*); (**b**) compressive strength (ST) and immersion pulverization rate ($\eta_{m}$); (**c**) specific surface area (BET) and water absorption rate (K); (**d**) pH, wetting angle $\left(\theta \right),$ and Zeta potential (ζ).

Fixing the amount of other agents, according to the threshold interval from wt 0.4% to wt 8.5% of H_2_O_2_ in Table 6, from small to large in accordance with a certain step size, we take 150 points into the optimized BP neural network for the calculation and inverse normalization and fit the synthetic curves as shown in Figure 6. As can be seen from Figure 6a–d, when the dose of H_2_O_2_ was 0.4–2%, the adsorption capacity of the adsorbent on total phosphorus increased from 35.13 mg·g^−1^ to 44.67 mg·g^−1^, and the removal rate increased from 68.72% to 87.38%, but the strength decreased from 1.32 kPa to 0.92 kPa. Further, the water-immersion pulverization rate of the particles decreased, and the soaking pulverization rate increased from 1.5% to 4.2%. The oxidation and foaming effects of H_2_O_2_ led to a rapid increase in the specific surface area of the adsorbent. When the dose of H_2_O_2_ increased from 0.4% to 2.0%, the specific surface area of the adsorbent increased from 23.95 m^2^·g^−1^ to 35.23 m^2^·g^−1^; thus, the water absorption rate also increased because of the formation of more pores. With an increase in H_2_O_2_, some basic substances were oxidized [23], and the pH value of RM tended to decrease; furthermore, the surface tension decreased, and the surface energy increased. The liquid formed a continuous phase on the solid surface, the wetting angle became smaller, and the Zeta potential changed in the positive direction, which was beneficial for the adsorption of anionic pollutants.

#### 2.2.4. Effect of HCl on Adsorbent Properties

**Figure 7:**
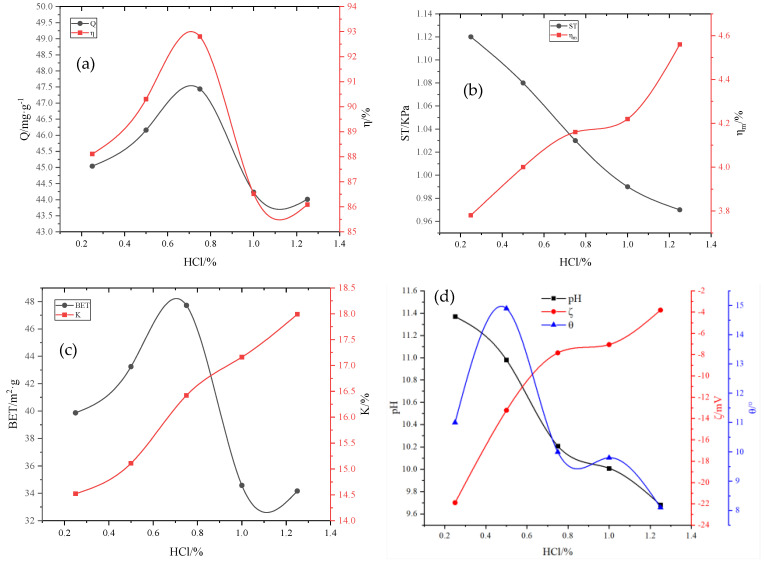
Influence of HCl on various adsorbent properties: (**a**) adsorption capacity (Q) and removal rate (*η*); (**b**) compressive strength (ST) and immersion pulverization rate ($\eta_{m}$); (**c**) specific surface area (BET) and water absorption rate (K); (**d**) pH, wetting angle $\left(\theta \right),$ and Zeta potential (ζ).

Calculating the properties of the adsorbent with the addition of hydrochloric acid using the same method as mentioned above, the properties of the HCl-modified particle adsorbents are shown in Figure 7a–d. After adding H_2_O_2_ and MnO_2_ foaming agents, HCl was added to activate the adsorbent, which further improved the porosity of the adsorbent [26]. When the dose of HCl was 0.75%, the adsorption capacity of total phosphorus was 47.44 mg·g^−1^, and the removal rate was 92.80%. The strength was 1.03 kPa, and the 24-h immersion pulverization rate was 4.16%. HCl is a strong inorganic acid and can release a large amount of H^+^ in the binder. With an increase in the HCl dose, the adsorption capacity of the adsorbent first increased and then decreased, and the adsorption effect was significant when 0.75% HCL was added. Thereafter, as the dose of HCl was further increased, the adsorption capacity and strength decreased, and the immersion pulverization rate increased. When HCl content exceeds 0.75%, OH^−^ and H^+^ in the system are neutralized by acid and base, respectively, resulting in increased pores and partially forming through pores, which increases the desorption capacity of the system and decreases the adsorption capacity. When HCl was added, the water absorption increased with an increase in HCl dose, but the specific surface area increased initially and then decreased [27,28]. When the dose of HCl was 0.75%, the specific surface area of the adsorbent increased to 47.72 m^2^·g^−1^. Thereafter, as the dose of HCl further increased, the specific surface area began to decrease. The addition of HCl introduced a large amount of H^+^, reducing the pH, and the wetting angle increased initially and then decreased, and the Zeta potential changed in the positive direction, which was conducive to the adsorption of anions.

This is because the H^+^ in HCl is more active. In the process of granulation, HCl dissolves k^+^, Ca^2+^, Na^+^, Fe^2+^, and Al^3+^ in the adsorbent, eliminating the original interlayer bonding force. The laminar crystal lattice is cracked [22]. Coupled with the fact that after the activation of the HCl-activated mineral-like substances, there is a change in the structure of the pores and the voids, and the acid removes impurities from them, so the pore channels are unclogged, which is favorable for the Pollutant molecules to enter and adsorb. When the amount of addition increases, the OH^−^ and H^+^ in the system for acid–base neutralization, the pore further increase. Part of the formation of through holes increases the desorption capacity of the system, reducing the adsorption capacity. At this time, the adsorbent is relatively flimsy, the strength of the adsorbent is reduced, and the rate of leaching chalking is increased [22].

#### 2.2.5. Effect of the Surfactant on the Adsorbent Properties

**Figure 8:**
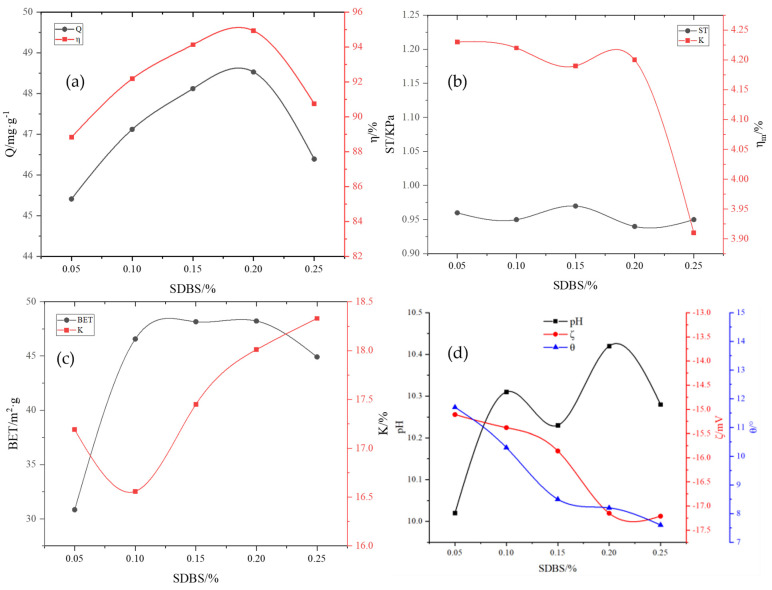
Influence of dodecylbenzene sulfonate (SDBS) on various adsorbent properties: (**a**) adsorption capacity (Q) and removal rate (*η*); (**b**) compressive strength (ST) and immersion pulverization rate ($\eta_{m}$); (**c**) specific surface area (BET) and water absorption rate (K); (**d**) pH, wetting angle $\left(\theta \right),$ and Zeta potential (ζ).

The adsorbent properties after the addition of surfactant SDBS are shown in Figure 8a–d. When the SDBS dose was increased from 0.05% to 0.25%, the adsorption capacity and removal rate of total phosphorus first increased and then decreased. The adsorption performance was reduced as the amount of SDBS was increased due to the formation of wide pores caused by the increased foaming of microbubbles. When the SDBS dose was 0.2%, the adsorption capacity of total phosphorus reached 48.53 mg·g^−1^, and the removal rate reached 94.93%. SDBS is an anionic surfactant with a certain foaming effect. The addition of SDBS led to a further increase in the micropores, specific surface area, surface activity, and water absorption and resulted in good hydrophilicity. On the contrary, it caused a decrease in the surface wetting angle. With the addition of SDBS, the pH of the adsorbent fluctuated within a small range; the wetting angle decreased with an increase in the SDBS dose, and the Zeta potential changed in the negative direction with an increase in the SDBS dose. Furthermore, the adsorbent became stable.

### 2.3. Preparation and Test of the Active RM Adsorbent with the Optimal Formulation

After the optimization ratio calculation is completed, the adsorbent is prepared according to the optimized formulation, and the performance of the adsorbent is verified to determine the optimized formulation finally. H_2_O_2_, HCl, and SDBS were found to be the important agents affecting adsorption capacity. The synergistic effect of these three agents increased the specific surface area of the adsorbent, reduced the wetting angle, and changed the wettability of the surface. The key agents affecting the strength were HPMC and aluminate cement, whose synergistic effect enhanced the compressive strength and water-immersion pulverization rate. However, during non-thermal activation, there is a tradeoff between the regulation of adsorption capacity and mechanical properties. The evaluation results of the BP neural network can better guide the optimization of the adsorbent formulation in the field.

Table 7 lists the optimization results of the outputs. The formulation of RM particle adsorbents is as follows: According to the following mass ratio, the primary material (RM 95% + FA 5.2%), admixture (water–cement ratio 0.33 + cement 8.2% + HPMC 0.25% + sodium silicate 2% + coupling agent 0.01% + H_2_O_2_ 1.64% + MnO_2_ 0.33% + HCl 0.75% (concentration: 37%) + SDBS 0.21%) and 9% nucleated particle proppant were mixed to prepare 30 kg of RM particle adsorbents, with a pH of 10.16, the specific surface area of 48.92 m^2^·g^−1^, pore volume of 2.10 cm^3^·g^−1^, compressive strength of 1.12 kPa, 24 h pulverization rate of 3.72%, and true density of 1530 kg·m^−3^. The prepared RM particle adsorbents were used to adsorb the total phosphorus in recycling water continuing flotation tailings. When the initial concentration of total phosphorus was 127.8 mg·L^−1^, the amount of RM particle adsorbents was 25 g·L^−1^, and the adsorption time was 14 h, and the adsorption capacity and removal rate of total phosphorus were 48.63 mg·g^−1^ and 95.13%, respectively. 

## 3. Materials and Methods

### 3.1. Test Materials

RM, fly ash (FA), aluminate cement (A2C), and manganese dioxide (MnO_2_) are the main raw materials of the particle adsorbents in this study. The moisture content of RM was 30%. After drying at 50 °C for 12 h, the RM was ground by a ball mill and screened using a 0.075 mm sieve. For FA, the burning loss was ≤5%, SiO_2_ content was ≥30%, and SO_3_ content was ≤2%; for the aluminate cement, Al_2_O_3_ accounted for 50%; CaO, 30%; SiO_2_, 10%; and Fe_2_O_3_, 3%. MnO_2_ was of industrial grade, with a purity of 97%.

The main reagents used in the test were hydrogen peroxide (H_2_O_2_), HCl (concentration 36–38%), ICP test standard solution, HNO_3_ (concentration 68%), sodium dodecyl benzene sulfonate (SDSB), and sodium silicate (Na_2_SiO_4_), all of which were analytically pure. Furthermore, commercially pure hydroxypropyl methylcellulose (referred to as HPMC, 2 million mPa·s) and KH-602 silane-coupling agent were used. The adsorption activity site of the adsorbent was improved by adding FA [29,30], which was rich in Fe and Al compounds and larger than the surface. Foaming catalysts like H_2_O_2_ [31] and MnO_2_ [32] were added to increase the specific surface area of the adsorbent. The pH value, Zeta potential, and pore size distribution of the adsorbent were changed due to HCl [33] activation. The anionic surfactant and wetting agent sodium dodecylbenzene sulfonate (SDBS) [34] were used to alter the adsorbent’s wettability and surface activity, while also increasing its adsorption capacity. Combining the reinforcing agent (A2C + HPMC + Na_2_SiO_4_ + KH-602) enhanced the adsorbent’s strength and immersion loss rate [35,36].

For the test, phosphorus-containing wastewater was collected from a phosphate ore dressing plant in Guizhou, China. The recycling water containing flotation tailings was collected; this water contained 1278 mg·L^−1^ of phosphorous. To prepare RM particle adsorbents, the recycling water containing flotation tailings diluted 10 times was selected as the test water. This choice is based on the measurement range of the ICP instrument (Thermo Fisher Scientific, Waltham, MA, USA, model ICP-7400). 

The test equipment included a PQ10 granulating disk, electronic balance (model: BL-2000F), ball mill (XMGB Φ 305 × 3.5), SHBY-40B constant temperature and humidity standard curing box, SHBY-40B water bath thermostatic oscillator, JJ-1 enhanced electric agitator, HY-4 speed-regulating multipurpose oscillator, and self-made dynamic adsorption and desorption integrated equipment.

### 3.2. Preparation Method of the RM Adsorbent

Preparation of the main material for the adsorbent powder: RM and aluminate cement were added to the agitator with a mass ratio of 6:4. After mixing the materials evenly, the mixture was placed in a granulating disk, and deionized water was sprayed into the agitator while rotating until the mixture was formed. Subsequently, a 1 mm sieve was used to remove circular particles. After sieving, the mixture was dried in a 40 °C electric blast-drying oven till the water content was less than 5%, resulting in the formation of nucleating granule support. In the high-speed powder-dispersing machine, the adsorbent powder was prepared by adding RM, 1.7–8.5% FA, 2–10% A2C, 10–20% MnO_2_, and other powders according to the ratio.

Preparation of the active binder: KH-602, hydroxypropyl methylcellulose (HPMC), and Na_2_SiO_4_ powders were added into the deionized water with a water–binder ratio of 3:1, with mass ratios of 0.1–0.4%, 0.5–2.5%, and 2–4%, respectively. Thereafter, the mixture was magnetically stirred for 5 min, and then it was kept standing for ~24 h for later use; subsequently, H_2_O_2_ was added according to the mass ratio of 0.4–2.0%, followed by magnetic stirring for 5 min and standing for ~24 h; HCl and SDBS were added with mass ratios of 0.25–1.2% and 0.05–0.25%, respectively, followed by magnetic stirring for 5 min and standing for ~24 h, resulting in the formation of the active binder.

Preparation of RM particle adsorbents: First, 10–20% nucleating particle support was added to the granulating disk, and the adsorbent powder was added while rotating. The active binder was slowly sprayed in continuously till the adsorptive active coating attained a certain thickness. After rotating for another 10 min, the adsorbent was screened and placed in the cement concrete standard curing box under constant temperature and humidity for 3 days and finally dried at 40 °C in the electric blast-drying oven. Thus, the RM particle adsorbents were obtained.

The purpose of this study is to build a neural network model using the limited experimental data available from the experiment and then predict more data points by the model, so the experiment mainly obtains limited data points. The model can achieve non-linear interpolation through the relationship of these data points; the input training data of the network are single-factor test data and orthogonal test data of the RM particle adsorbents, and the output data are performance test data of non-thermal activated RM particle adsorbents. In all, 131 sets of training data were used. Among them, the first 31 groups of training data were single-factor test data. In the single-factor test, 300 g of RM with a water–cement ratio of 1:3, 2% Na_2_SiO_4_, and 0.1% KH-602 were used as blank tests. They were added as indicated in Table 8. After the #02 to #06 tests, the amount of RM added was set to 285 g, and the FA concentration was set to 5.1%. Then, the concentrations of A2C, HPMC, H_2_O_2_, MnO_2_, and HCl were determined as 8%, 0.25%, 1.6%, 0.32%, and 0.75%, respectively. The last 100 groups of training data were 11-factor 5-level orthogonal test data, where the concentrations of aluminate cement and Na_2_SiO_4_ were determined as 8% and 2%, respectively. Then, the orthogonal test was performed by changing the amount of RM, FA, H_2_O_2_, MnO_2_, HPMC, HCl, and SDBS, with a water–cement ratio of 1:3.

In order to obtain the ideal BP neural network prediction model, there must be a sufficient number of input and output data pairs. In this study, a total of 131 adsorbents of red mud particles were prepared by arranging one-way and orthogonal tests based on the input and output data pairs of the BP neural network and formulation requirements.

### 3.3. Performance Tests of the RM Adsorbent

After the preparation of the adsorbent, it is necessary to test nine properties—specific surface area (BET), wetting angle (θ), Zeta potential (ζ), adsorption capacity (Q) [27], compressive strength (ST) [37], removal rate ($\eta_{m}$) [38], immersion pulverization rate(K), water absorption rate, and pH—of the adsorbent, of which the adsorption capacity, compressive strength, removal rate, and pulverization rate are the important basic indexes to measure the particle adsorbents. Zeta potential [39] is a useful metric for determining the adsorption system’s stability and electrochemical properties. Colloids with high Zeta potential are stable, while those with low Zeta potential tend to flocculate. The pH value [40] is the main influencing factor of Zeta potential. There is a good correlation between wettability and adsorption capacity, and the better the wettability [41] of the adsorbent surface, the better the adsorption effect. The larger specific surface area [42] is beneficial to the adsorption effect of the adsorbent, and the specific surface area is related to the water absorption [43] and adsorption capacity.

The removal rate *η* of phosphorus in the solution, the adsorption capacity Q of the adsorbent, the pulverization rate of the adsorbent $\eta_{m}$, and the water absorption rate K of the adsorbent were calculated according to Equations (1) and (4) as follows:

(1) $$\eta =\frac{C_{0}-C_{i}}{C_{0}}\times 100\%$$

(2) $$Q=\frac{V(C_{0}-C_{i})}{m}\times 100\%$$

(3) $$\eta_{m}=\frac{m-m_{i}-m_{s}}{m}\times 100\%$$

(4) $$K=\frac{m_{1}-m}{m}\times 100\%$$

where *η* (%) represents the removal rate of phosphorus in the solution; $C_{0}$ (mg·L^−1^) represents the concentration of phosphorus in the solution before adsorption; $C_{i}$ (mg·L^−1^) represents the concentration of phosphorus in the solution after adsorption; Q (mg·g^−1^) represents the adsorption capacity of adsorbent per unit mass; V (L) represents the volume of the solution; m (g) represents the mass of the adsorbent after drying; $\eta_{m}$ (%) represents the pulverization rate of the adsorbent; $m_{i}$ (g) represents the drying mass of the adsorbent after adsorption; $m_{s}$ (g) represents adsorbing capacity; K (%) represents the water absorption rate; $m_{1}$ (g) represents the mass of deionized water absorbed by the adsorbent.

Micro metrics measured the specific surface area of particle adsorbents ASAP2020 N2 adsorption/desorption physical adsorption instrument (BET), and the specific surface area (S_BET_) was calculated using the Brunauer–Emmett–Teller method.

The Zeta potential (ζ) on the surface of the adsorbent before and after adsorption was determined using the nanoparticle size/Zeta potential analyzer (Beckman Coulter, Pasadena, CA, USA, model: DELSA Nano C). The test sample was put into a beaker, and a certain volume of deionized water was added. The suspension was prepared with a water–cement mass ratio of 100:1. After magnetic stirring for 10 min, the suspension was fully dispersed and then left standing for 6 h. Subsequently, the supernatant was collected into the sample cell and placed in a potentiometer to determine the Zeta potential.

The wetting angle of the particle adsorbents was measured by the LAUDA wetting angle measuring instrument/contact angle measuring instrument (LAUDA Scientific, GmbH, Germany, model: LSA60). Since a block sample reflects the real wetting angle of the mineral better than a powder-pressed sample, 1 × 1 × 1 cm cubic mineral blocks that were ground with sandpaper, washed with distilled water, and dried naturally before being used were adopted for all the wetting angle tests. An ore cube was immersed in the solution to be tested for 30 min and then taken out to measure the wetting angle by the floating bubble method. The measurement was repeated five times to obtain the average value.

Particle strength ST (KPa) was measured by the APT-3 particle strength tester.

The pH value of the solution is affected by the content of the adsorbent formulation. The pH value of the phosphorus-containing wastewater after adsorption is measured using a pH meter.

### 3.4. Establishment and Application of the BP Neural Network Model

The main objective of adsorbent formulation optimization was to improve the mechanical adsorbent properties while satisfying the adsorption capacity. The preparation conditions clearly indicate that there exists certain coupling and nonlinearity among the various adsorbent properties, and the orthogonal test method cannot satisfy the formulation optimization. Therefore, the neural network was used for nonlinear formulation optimization to obtain the relationship between the appropriate ratio and the optimal performance.

#### 3.4.1. Determination of Input and Output Training Datasets

The design of 131 red mud adsorbent formulations according to Section 3.2 was used as input data for the BP neural network, and the performance tested in Section 3.3 was used as output data. When the input value is too large, the neurons will be in a saturated state, thus losing the ability to learn. Therefore, the input value should be normalized, and the input value should be processed from 0 to 1. The normalized function is shown in Equation (5) [44].

(5) $$x^{\prime }=(x-x_{\min})/(x_{\max}-x_{\min})$$

The input and output data sets were normalized. Similarly, the predicted results were normalized and then output. The training parameters were set after normalizing the training data. Among the 131 experimental data, 84 data were randomly selected as training data for neural network training and 47 data were used as testing data for network testing. 

#### 3.4.2. Establishment of a BP Network Optimization Model

**Figure 9:**
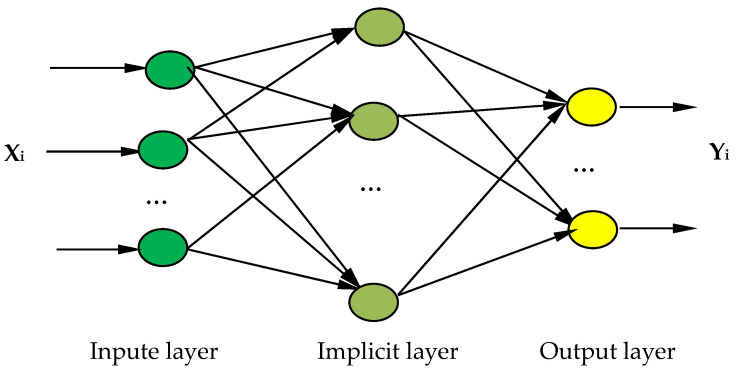
Back-propagation (BP) network optimization model for the formulation optimization of the RM particle adsorbents.

The BP neural network optimization model of the nonthermally active RM particle adsorbents is shown in Figure 9. The model has an 11-input-9-output structure. The designed neural network has a three-layered structure, including the input layer, output layer, and hidden layer, where X is the input layer, representing the parameter change of each formulation. Y represents the amount of change in each performance index caused by a change in X and is used to investigate the influence of the change amount of each factor on the performance index of the adsorbent. The following formulation was used to select the number of neurons in the hidden layer in Equation (6) [44].

(6) $$l=\sqrt{m+n}+\alpha$$

where m represents the number of nodes in the input layer; l represents the number of nodes in the hidden layer; n represents the number of nodes in the output layer; and α is a constant between 1 and 10; in this paper,  m = 11,  n = 9, α= 10, l = 14.

The input–output relationship is represented as follows:

X = {RM, FA, Water–Cement Ratio, A2C, HPMC, Na_2_SiO_4_, KH-602, H_2_O_2_, MnO_2_, HCl, SDBS}, 

Y = {specific surface area, wetting angle, Zeta potential, adsorption capacity, removal rate, compressive strength, immersion pulverization rate, water absorption rate, pH}.

The output layer transfer function chooses the linear function. In this paper, the Sigmoid function is selected as an intermediate layer transfer function, and the training strategy is a gradient reduction method [45].

#### 3.4.3. Training and Testing

While the BP neural network model is built, the network is programmed and trained, and MATLAB R2022b software was used to set parameters as follows: numberOfSample is 84, numberOfTestSample is 47, numberOfForcastSample is 9, numberOfHiddenNeure is 14, inputDimension is 11, outputDimension is 9, net.trainParam.show is 10, net.trainParam.epochs is 10,000, net.trainParam.lr is 0.035, net.trainParam.goal is 10^−3^, and net.divideFcn is ‘’. After the network is trained, the network is tested with 47 sets of test samples, and the testing data are given in Table 3. The network is evaluated using mean square error (MSE, Equation (7)), root mean square error (RMSE, Equation (8)), and coefficient of determination (R^2^, Equation (9)) [46].

(7) $$MSE=\frac{1}{n}\sum_{i=0}^{n}{\left(\hat{y_{i}}-y_{i}\right)}^{2}$$

(8) $$RMSE=\sqrt{\frac{1}{n}\sum_{i=0}^{n}{\left(\hat{y_{i}}-y_{i}\right)}^{2}}$$

(9) $$R^{2}=1-\frac{\frac{1}{n}\sum_{i=0}^{n-1}{\left(\hat{y_{i}}-y_{i}\right)}^{2}}{dx\frac{1}{n}\sum_{i=0}^{n-1}{\left(\bar{y}-y_{i}\right)}^{2}}$$

where *n* is the number of input/output data pairs; $\hat{y_{i}}$ is the predicted value; $y_{i}$ is the experimental value; and $\bar{y}$ is the average of predicted values.

#### 3.4.4. Formulation Optimization

While the network has been trained and tested, the model can be used to predict and analyze the results of the experiment [44]. During formulation optimization, 11 input parameters and 9 output parameters were optimized. By fixing the ratio of 11 agents and changing the dosage of the agent to be optimized, the value was calculated according to the range of the dosage from small to large with a certain step size, and the changes in the output parameters at each change point were compared. Then, according to the target output value of the RM particle adsorbents, the optimal ratio was derived. The range of the optimized parameters is shown in Table 6.

## 4. Conclusions and Future

(1) A novel approach to optimize the formulation of RM adsorbent by establishing a BP neural network model is proposed. From the single-factor test and orthogonal test, learning samples of the BP neural network were obtained, thereby reducing the number of experiments and ensuring the representativeness of the learning samples. The application of the BP neural network compensates for the limitation of the orthogonal test and simulates and predicts the strength and adsorption capacity of RM adsorbent over a wide range to obtain the optimal formulation of the adsorbent.
(2) After formulation optimization based on the BP neural network, granulation was performed according to the preparation and curing method of RM particle adsorbents, and the adsorbent was prepared with a pH of 10.16, specific surface area of 48.92 m^2^·g^−1^, pore volume of 2.10 cm^3^·g^−1^, compressive strength of 1.12 kPa, and 24 h immersion pulverization rate of 3.72%. The prepared adsorbent was used to adsorb total phosphorus from phosphorus-containing wastewater. When the initial concentration of total phosphorus was 127.8 mg·L^−1^, the amount of the adsorbent was 25 g·L^−1^ and the adsorption time was 14 h. The adsorption capacity and removal rate of total phosphorus were 48.63 mg·g^−1^ and 95.13%, respectively, indicating a significant phosphorus removal effect.
(3) The verification results prove that the prediction results of the established prediction model are reliable, and it is feasible and effective to predict the strength and adsorption capacity and optimize the formulation of RM adsorbent via the BP neural network, which provides reference and scientific guidance for the experimental design of RM adsorbent. However, the application of neural networks in the optimization of material formulation is still in the exploratory stage, and further study and improvement are required.

In this study, although success was achieved in multi-input and multi-output recipe optimization using BP neural networks, the experimental data points are still insufficient and the coupling relationship and interactions between parameters need to be further investigated.

## Figures and Tables

**Table 1:** Partial input training data.

No.	RM/g	FA/%	Water–Cement	A2C/%	HPMC/%	Na_2_SiO_4_/%	KH-602/%	H_2_O_2_/%	MnO_2_/%	HCl/%	SDBS/%
1	285	5.1	1:3	8	0.25	2	0.1	0	0	0	0
2	285	5.1	1:3	8	0.25	2	0.1	0.4	0.08	0	0
3	285	5.1	1:3	8	0.25	2	0.1	0.8	0.16	0	0
4	285	5.1	1:3	8	0.25	2	0.1	1.2	0.24	0	0
5	285	5.1	1:3	8	0.25	2	0.1	1.6	0.32	0	0
6	285	5.1	1:3	8	0.25	2	0.1	2.0	0.4	0	0
7	285	5.1	1:3	8	0.25	2	0.1	1.6	0.32	0.25	0
8	285	5.1	1:3	8	0.25	2	0.1	1.6	0.32	0.5	0
9	285	5.1	1:3	8	0.25	2	0.1	1.6	0.32	0.75	0
10	285	5.1	1:3	8	0.25	2	0.1	1.6	0.32	1	0
11	285	5.1	1:3	8	0.25	2	0.1	1.6	0.32	1.25	0
12	285	5.1	1:3	8	0.25	2	0.1	1.6	0.32	0.75	0
13	285	5.1	1:3	8	0.25	2	0.1	1.6	0.32	0.75	0.05
14	285	5.1	1:3	8	0.25	2	0.1	1.6	0.32	0.75	0.1
15	285	5.1	1:3	8	0.25	2	0.1	1.6	0.32	0.75	0.15
16	285	5.1	1:3	8	0.25	2	0.1	1.6	0.32	0.75	0.2

**Table 2:** Partial output training data.

No.	BET/m^2^·g	θ/°	ζ/mV	Q/mg·g^−1^	*η*/%	ST/KPa	$\eta_{m}$/%	K/%	pH
1	19.28	18.9	−18.98	25.98	50.82	1.46	1.20	6.46	11.69
2	23.95	10.6	−8.01	35.13	68.72	1.32	1.50	8.42	11.55
3	25.79	10.6	−7.98	38.59	75.49	1.28	1.60	9.18	11.53
4	29.66	9.1	−6.47	42.13	82.41	1.21	2.70	11.47	11.49
5	31.45	6.6	−5.62	43.52	85.13	1.11	3.50	12.32	11.56
6	35.23	5.5	−5.41	44.67	87.38	0.92	4.20	14.48	11.43
7	39.87	11.0	−21.9	45.04	88.11	1.12	3.78	14.52	11.37
8	43.24	14.9	−13.23	46.16	90.30	1.08	4.00	15.11	10.98
9	47.72	10.0	−7.83	47.44	92.80	1.03	4.16	16.42	10.21
10	34.58	9.8	−7.05	44.23	86.52	0.99	4.22	17.16	10.01
11	34.17	8.1	−3.81	44.01	86.09	0.97	4.56	17.99	9.68
12	29.76	14.4	−13.44	42.44	83.02	1.22	4.15	16.23	10.24
13	30.84	11.7	−15.11	45.41	88.83	0.96	4.23	17.19	10.02
14	46.57	10.3	−15.38	47.12	92.18	0.95	4.22	16.56	10.31
15	48.16	8.5	−15.86	48.12	94.13	0.97	4.19	17.45	10.23
16	48.23	8.2	−17.15	48.53	94.93	0.94	4.20	18.01	10.42

**Table 3:** Partial input testing data.

No.	RM/g	FA/%	Water–Cement	A2C/%	HPMC/%	Na_2_SiO_4_/%	KH-602/%	H_2_O_2_/%	MnO_2_/%	HCl/%	SDBS/%
1	285	5.1	1:3	8	0.25	2	0.1	1.6	0.32	0.75	0.25
2	285	5.1	1:3	8	0.25	2	0.1	0.8	0.16	0.25	0.10
3	285	5.1	1:3	8	0.25	2	0.1	0.8	0.16	0.50	0.15
4	285	5.1	1:3	8	0.25	2	0.1	0.8	0.16	0.75	0.20
5	285	5.1	1:3	8	0.25	2	0.1	0.8	0.16	1.00	0.25
6	285	5.1	1:3	8	0.25	2	0.1	1.2	0.24	0.25	0.20
7	285	5.1	1:3	8	0.25	2	0.1	1.2	0.24	0.50	0.25
8	285	5.1	1:3	8	0.25	2	0.1	1.2	0.24	0.75	0.10
9	285	5.1	1:3	8	0.25	2	0.1	1.2	0.24	1.00	0.15
10	285	5.1	1:3	8	0.25	2	0.1	1.6	0.32	0.25	0.25
11	285	5.1	1:3	8	0.25	2	0.1	1.6	0.32	0.50	0.20
12	285	5.1	1:3	8	0.25	2	0.1	1.6	0.32	0.75	0.15
13	285	5.1	1:3	8	0.25	2	0.1	1.6	0.32	1.00	0.10
14	285	5.1	1:3	8	0.25	2	0.1	2.0	0.40	0.25	0.15
15	285	5.1	1:3	8	0.25	2	0.1	2.0	0.40	0.50	0.10
16	285	5.1	1:3	8	0.25	2	0.1	2.0	0.40	0.75	0.25

**Table 4:** Partial output testing values.

No.	BET/m^2^·g	θ/°	ζ/mV	Q/mg·g^−1^	*η*/%	ST/KPa	$\eta_{m}$/%	K/%	pH
1	43.239	84.591	0.930	3.842	42.240	16.240	11.420	−16.430	7.900
2	44.450	86.962	0.960	3.867	41.231	17.130	10.980	−16.120	7.400
3	43.169	84.453	0.920	3.772	41.319	17.210	10.290	−15.340	7.400
4	44.589	87.232	0.910	3.700	42.450	16.300	11.180	−15.390	7.500
5	46.120	90.229	0.940	3.886	44.560	15.980	10.880	−14.290	7.200
6	46.529	91.022	0.920	3.935	43.779	16.420	10.210	−14.280	7.100
7	47.117	92.175	0.930	4.018	43.390	16.890	10.170	−14.110	7.200
8	47.218	92.375	0.910	3.918	44.560	16.560	11.420	−14.270	7.300
9	47.317	92.576	0.960	3.649	45.110	17.110	10.980	−13.990	6.900
10	46.428	90.834	0.970	3.776	46.120	17.200	10.290	−13.720	6.800
11	48.360	94.621	0.980	4.027	47.295	17.250	10.705	−14.490	7.850
12	47.208	92.352	0.940	4.092	46.210	17.230	11.880	−13.430	7.100
13	48.438	94.754	0.920	3.630	47.480	17.500	10.210	−13.010	6.500
14	47.122	92.167	0.910	3.946	48.120	16.980	10.170	−12.410	6.400
15	47.586	93.097	0.880	3.959	48.251	17.230	10.120	−12.150	6.400
16	47.616	93.156	0.860	4.041	48.990	17.360	10.030	−11.070	6.300
RMSE	0.0033	0.0039	0.0031	0.0014	0.0120	0.0070	0.0891	0.0312	0.0200

**Table 5:** Experimental values corresponding to the testing data in Table 3.

No.	BET/m^2^·g	θ/°	ζ/mV	Q/mg·g^−1^	*η*/%	ST/KPa	$\eta_{m}$/%	K/%	pH
1	44.92	7.6	−17.21	46.39	90.75	0.95	3.91	18.33	10.28
2	42.24	7.9	−16.43	43.24	84.59	0.93	3.87	16.24	11.42
3	41.23	7.4	−16.12	44.45	86.96	0.96	3.92	17.13	10.98
4	41.32	7.40	−15.34	43.17	84.45	0.92	3.88	17.21	10.29
5	42.45	7.50	−15.39	44.59	87.23	0.91	3.67	16.30	11.18
6	44.56	7.20	−14.29	46.12	90.23	0.94	3.89	15.98	10.88
7	43.78	7.10	−14.28	46.53	91.02	0.92	3.95	16.42	10.21
8	43.39	7.20	−14.11	47.12	92.17	0.93	3.96	16.89	10.17
9	44.56	7.30	−14.27	47.22	92.37	0.91	3.97	16.56	11.42
10	45.11	6.90	−13.99	47.32	92.57	0.96	3.64	17.11	10.98
11	46.12	6.80	−13.72	46.43	90.83	0.97	3.72	17.20	10.29
12	46.43	7.20	−13.12	48.57	95.17	0.99	3.98	17.05	11.18
13	46.21	7.10	−13.43	47.21	92.35	0.94	3.99	17.23	11.88
14	47.48	6.50	−13.01	48.44	94.75	0.92	3.65	17.50	10.21
15	48.12	6.40	−12.41	47.12	92.17	0.91	3.94	16.98	10.17
16	48.25	6.40	−12.15	47.59	93.09	0.88	3.98	17.23	10.12

**Table 6:** The range of the optimized parameters.

Name of Component	RM/g	FA/%	Water–Cement	A2C/%	HPMC/%	Na_2_SiO_4_/%
Range	275–300	1.7–8.5	1:3	2–10	0.05–0.25	2
Name of component	KH-602/%	H_2_O_2_/%	MnO_2_/%	HCl/%	SDBS/%	
Range	0.1	0.4–2	0.08–0.4	0.25–1.25	0.05–0.25	

**Table 7:** Performance parameters of RM particle adsorbents.

**BET/m^2^·g^−1^**	**Zeta Potential/mV**	**Wetting Angle/°**	**Immersion Pulverization Rate/%**	**pH**
48.92	−8.23	6.1	3.79	10.16
**Adsorption capacity of phosphorus/mg·g^−1^**	**Removal rate of phosphorus/%**	**Compressive strength/KPa**	**Water absorption/%**	
48.63	95.13	1.12	16.58	

**Table 8:** Single-factor test data table.

#02–#06		#07–#11	#12–#16	#17–#21	#22–#26		#27–#31
RM/g	FA/%	A2C/%	HPMC/%	H_2_O_2_/%	MnO_2_/%	HCl/%	SDBS/%
295	1.7	2	0.05	0.4	0.08	0.25	0.05
290	3.4	4	0.10	0.8	0.16	0.5	0.1
285	5.1	6	0.15	1.2	0.24	0.75	0.15
280	6.8	8	0.20	1.6	0.32	1.00	0.2
275	8.5	10	0.25	2.0	0.4	1.25	0.25

## Data Availability

Data are contained within the article.

## Abbreviations

BP	Back-propagation neural networks	BET	Specific surface area	η	Removal rate
FA	Fly ash	θ	Wetting angle	K	Immersion pulverization rate
RM	Red mud	ζ	Zeta potential	$\eta_{m}$	Water absorption rate
SDBS	Sodium dodecylbenzene sulfonate	Q	Adsorption capacity		
A2C	Aluminate cement	ST	Compressive strength		
